# Pulse EPR Measurements of Intramolecular Distances in a TOPP-Labeled Transmembrane Peptide in Lipids

**DOI:** 10.1016/j.bpj.2016.10.022

**Published:** 2016-11-09

**Authors:** Karin Halbmair, Janine Wegner, Ulf Diederichsen, Marina Bennati

**Affiliations:** 1 Electron Spin Resonance Spectroscopy, Max Planck Institute for Biophysical Chemistry, Göttingen, Germany; 2 Institute for Organic and Biomolecular Chemistry, Georg-August-University, Göttingen, Germany

## Abstract

We present the performance of nanometer-range pulse electron paramagnetic resonance distance measurements (pulsed electron-electron double resonance/double electron-electron resonance, PELDOR/DEER) on a transmembrane WALP24 peptide labeled with the semirigid unnatural amino acid 4-(3,3,5,5-tetra-methyl-2,6-dioxo-4-oxylpiperazin-1-yl)-l-phenylglycine (TOPP). Distances reported by the TOPP label are compared to the ones reported by the more standard MTSSL spin label, commonly employed in protein studies. Using high-power pulse electron paramagnetic resonance spectroscopy at Q-band frequencies (34 GHz), we show that in contrast to MTSSL, our label reports one-peak, sharp (Δ*r* ≤ 0.4 nm) intramolecular distances. Orientational selectivity is not observed. When spin-labeled WALP24 was inserted in two representative lipid bilayers with different bilayer thickness, i.e., DMPC and POPC, the intramolecular distance reported by TOPP did not change with the bilayer environment. In contrast, the distance measured with MTSSL was strongly affected by the hydrophobic thickness of the lipid. The results demonstrate that the TOPP label is well suited to study the intrinsic structure of peptides immersed in lipids.

## Main Text

**Figure 1:**
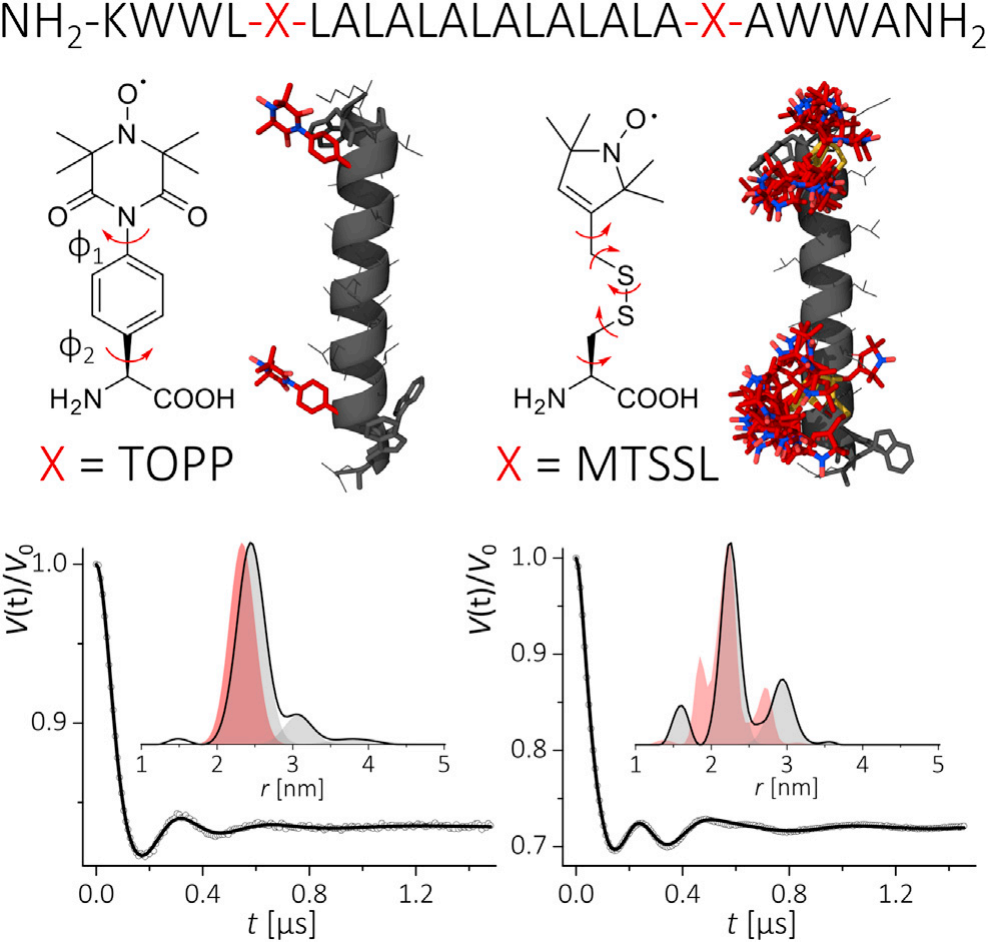
(*Top*) The peptide sequence of WALP24 showing the positions (X), at which the spin labels have been inserted. (*Center*) Chemical structure of the TOPP (*left*) and MTSSL (*right*) spin labels. Structures were modeled as explained in the Supporting Material. (*Bottom*) PELDOR experiments on WALP24 in methanol. Background-corrected PELDOR time traces (*dots*) and fits using Tikhonov regularization (DeerAnalysis) (26) (*lines*) for WALP24-TOPP (*left*) and WALP24-MTSSL (*right*). Experimental and modeled distance distributions are shown in comparison (*filled line and area*). Original traces are displayed in Fig. S3.

Understanding the functionality and organization of peptides and proteins in biological membranes requires knowledge of their molecular structures and conformational dynamics. Crystallization of membrane proteins or protein complexes is still challenging and conformations found in the crystallized state may not represent the biologically active species. Therefore, development of other complementary spectroscopic methods like nuclear magnetic resonance spectroscopy or electron paramagnetic resonance techniques (EPR) becomes essential (1, 2). Pulsed electron-electron double resonance (PELDOR) or double electron-electron resonance (DEER) is an EPR-based method that emerged as a powerful tool to measure interspin distances and orientations in biomolecules in a range between ∼2 and 10 nm (3, 4, 5). The method detects the magnetic dipole-dipole interaction between two paramagnetic centers, usually nitroxide radicals that are site-selectively inserted in biomolecules either via mutagenesis or more sophisticated methods such as ligation or synthetically generated peptides, or via in vivo introduction of unnatural amino acids (6, 7). In samples of peptides or proteins, intermolecular interactions can lead to high local concentrations of spin labels, which in turn reduce transverse spin relaxation and the detectable signal (8). In this case, the sensitivity of the PELDOR experiment becomes an issue. Performing distance measurements at Q-band frequencies and fields (34 GHz/1.2 T), a condition that is superior in terms of sensitivity over X-band (0.34 T, 9 GHz), becomes an essential advantage (9). Moreover, in lipid bilayers, the flexibility of standard labels such as MTSSL combined with nonhomogeneous distribution of peptides, leads to complex distance distributions. To overcome this issue, nitroxide labels with reduced mobility like RX and TOAC have been proposed in Schreier et al. (10) and Fleissner et al. (11). Although they have great potential, RX requires two cysteine mutations per spin label and TOAC is an achiral C*α*-bis substituted amino acid with impact on the peptide secondary structure. The latter is known to adopt helical torsion angle, rendering it well suited for investigating *β*-bends and *α*-helices while introduction into other structural motifs might cause structural distortions (10, 12, 13). As a potential alternative, we have previously introduced a semirigid spin label for peptide studies, called 4-(3,3,5,5-tetra-methyl-2,6-dioxo-4-oxylpiperazin-1-yl)-l-phenylglycine (TOPP) (Fig. 1, *inset*), based on an unnatural amino acid, in which the position of the spin bearing NO-group is fixed in space (7). We have reported the performance of TOPP in distance measurements in solution and for measurements of relative orientations with high-field EPR (14). Here we present the performance of TOPP as compared to the standard MTSSL label in reporting intramolecular distances in lipid environment.

As a model system for a comparative study, we have employed a WALP model peptide, which is composed of a hydrophobic stretch of alternating leucines and alanines flanked at both ends by a pair of tryptophans (15). The latter provide anchoring in the headgroup area of the membrane and are expected to influence the orientation of the peptide helix as a transmembrane segment. The rationale for the choice of WALP were the recent reports on the stability and good adaptation of WALP into lipid bilayers in conjunction with nitroxide spin labels and with more bulky labels such as lanthanide chelates (16, 17). Spin-labeled WALP24 peptides were synthesized by solid-phase peptide synthesis as described in Supporting Material. The amino acid sequence is shown in Fig. 1 (*top*) together with the labeling positions within the transmembrane domain of the peptide. While MTSSL was attached postsynthetically to a cysteine mutation at the selected label position, the TOPP amino acid was introduced by manual solid-phase peptide synthesis under conditions preventing racemization. Preservation of an *α*-helical structure of spin-labeled peptides in all environments used in the EPR experiments was confirmed by circular dichroism spectroscopy (Fig. S2). All distance measurements were performed using the standard four-pulse PELDOR sequence at Q-band frequencies with a high-power 170 W TWT amplifier (Supporting Material). Samples were of ∼20–30 *μ*M peptide bulk concentration in solvent or deuterated lipids.

To evaluate the capability of the TOPP label to report on intramolecular distances, we have first investigated a doubly labeled WALP24 peptide dissolved in methanol. In Fig. 1, typical PELDOR dipolar traces are shown that reflect the dipolar interaction between the two labels and their interspin distance. Dipolar oscillations are well visible in the traces recorded with both types of labels. However, inspection of the WALP24-MTSSL trace reveals the contribution of more than one dipolar frequency. Fourier transformation confirms the superposition of at least two Pake patterns in the latter case (Fig. S3). Instead, the trace recorded on the TOPP-labeled peptide shows one main frequency component. Analysis reveals a single-peak distance distribution for TOPP as compared to a more complex distribution with MTSSL. The observed distances and distributions could be well rationalized by simple molecular modeling as explained in Fig. S4 (18, 19). The predicted peak distance for the TOPP-labeled peptide (averaged among the O-O, N-O, and N-N distances) of *r* = 2.33 nm is in close agreement with the peak distance (most probable distance) from the experiment of *r* = (2.45 ± 0.05) nm and a distribution Δ*r* (peak half-width at half-height) of 0.2 nm. The small shift between the modeled and experimental peak distance (Δ ≈ 0.1 nm) actually exceeds the estimated experimental error (Fig. S3) and is likely due to simplicity of the structural modeling. The modeling clearly predicts that rotamers of MTSSL are responsible for the distance distribution with multiple peaks (Fig. 1). The results indicate that detailed interpretation of interspin distances using MTSSL labels becomes more difficult if distances arising from different conformations of the biomolecule might superimpose upon distances from MTSSL rotamers. Because the TOPP label is quasirigid, attention must be paid to whether the observed intramolecular distances and distributions are affected by orientation selection (14). To examine this, we have recorded the traces under the conditions of Fig. 1 but changing the resonance positions of detecting frequencies in the EPR line. A comparison of the resulting traces (Figs. S5–S7) reveals that there is no dependence of the dipolar frequency on the experimental setup, thus no orientation selection is observed.

**Figure 2:**
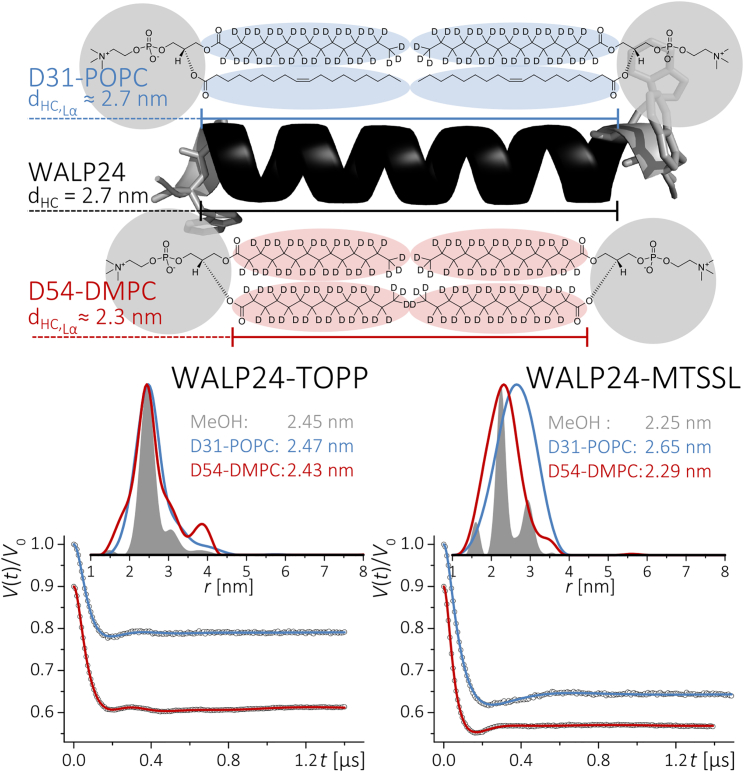
(*Top*) Chemical structure and schematic representation of the deuterated phospholipids D54-DMPC (14:0_2_-d54 PC) and D31-POPC (16:0-d31-18:1 PC) used in this study with their hydrophobic thickness, as compared to the length of WALP24. (*Bottom*) PELDOR experiments of TOPP- (*left*) and MTSSL-labeled (*right*) WALP24 in different environments. Comparison of dipolar traces after background subtraction and distance distribution obtained from fits using Tikhonov regularization (DeerAnalysis). Differences in modulation depth are due to labeling efficiency. Original traces are displayed in Fig. S10.

In a subsequent step, we have investigated the spin-labeled WALP24 peptide in two different representative lipids, DMPC and POPC. The hydrophobic length of WALP24 matches well the hydrophobic thickness of POPC (*r* ≈ 2.7 nm) but does not match the hydrophobic thickness of DMPC (*r* ≈ 2.3 nm), as sketched in Fig. 2. Therefore, these two lipids appeared as suited model systems to compare the capability of the labels to report on peptide structure in different lipid environments. To optimize the sample preparation for lipid studies, first an extensive study on WALP24-MTSSL in DMPC was performed. Different peptide/lipid values from 1:250 up to 1:3000 were tested to qualitatively monitor possible aggregation effects (Fig. S8). Aggregation of the spin-labeled peptide increases the number of spins participating in the PELDOR experiment, a fact that is manifested in an increasing modulation depth as well as in a faster spin-spin relaxation time *T*_2_ (20, 21). We have observed that for the ratio of 1:250 the *T*_2_ relaxation time (Fig. S9) and the background decay of the PELDOR signal (Fig. S8) were both drastically reduced. For a peptide/lipid ratio of 1:1500, *T*_2_ was shorter (Fig. S9) and the modulation depth increased (Fig. S8) as compared to the solution state, suggesting the onset of some aggregation. Therefore, to minimize intermolecular contributions, all distance measurements were performed at ratios at ∼1:3000 or even lower. Sample preparation was further optimized by using deuterated lipids (D31-POPC, D54-DMPC; Fig. 2) permitting us to prolong electron spin relaxation times. Fig. 2 displays a comparison of PELDOR traces for TOPP- and MTSSL-WALP24 in DMPC and POPC. As the most significant result, we have found that the two spin labels report significantly different distances. The peak distance between the TOPP labels does not show much dependence on the lipid environment within the experimental error, except for a slightly larger distribution up to Δ*r* = ± 0.4 nm in lipids as compared to methanol solution.

The result indicates that the peptide maintains its conformation in the two lipids and, at the same time, that TOPP is capable to report on the intrinsic peptide conformation. In contrast, the more flexible MTSSL label reports broad distance distributions. It is striking that the peak distances correspond to the hydrophobic thicknesses of the corresponding lipid. It has been reported that, due to their hydrophobicity, nitroxide spin labels insert into lipids and have a tendency to move to the interface region between the lipids tails and headgroups (22, 23). Thus, flexibility of MTSSL allows for adapting to the membrane thickness resulting in loss of information about the internal peptide structure. It has been proposed that tryptophan anchors can influence bilayer thickness (24), making the adaption of the bilayer one reasonable mechanism to react on a mismatch condition. However, these results with WALP24-MTSSL instead point to peptide tilting as an alternative adaptation mechanism that would not alter the interspin distance (2). A rigid label like TOPP will be ideally suited for the investigation of the peptide tilt angles by PELDOR spectroscopy in an aligned membrane (25). Overall, this study demonstrates that the TOPP label is well suited for high-resolution measurements of interspin distances in transmembrane peptides.

## Author Contributions

K.H. designed, performed, and analyzed EPR experiments; J.W. designed and performed peptide synthesis and characterization; M.B. helped with designing EPR experiments and wrote the article; and U.D. helped with designing peptide experiments and wrote the article.

## Supporting Material

Document S1. Supporting Materials and Methods and Figs. S1–S10

Document S2. Article plus Supporting Material
