# Supplementary material for: Pulse EPR Measurements of Intramolecular Distances in a TOPP-Labeled Transmembrane Peptide in Lipids
Source: Biophys J. 2016 Nov 9;111(11):2345–8. doi: 10.1016/j.bpj.2016.10.022 (PMC5153538; doi:10.1016/j.bpj.2016.10.022)
Supplement: Document S1. Supporting Materials and Methods and Figs. S1–S10 [file mmc1.pdf]

Biophysical Journal, Volume *111*

Supplemental Information

Pulse EPR Measurements of Intramolecular Distances in a TOPP-Labeled Transmembrane Peptide in Lipids

Karin Halbmair, Janine Wegner, Ulf Diederichsen, and Marina Bennati

Measurements of intramolecular distances in a TOPP-labeled transmembrane peptide in lipids by pulsed EPR spectroscopy

Supporting Information

Karin Halbmair,[1] Janine Wegner,[2] Ulf Diederichsen,[2] and Marina Bennati[1,2]

[1]Electron Spin Resonance, Max Planck Institute for Biophysical Chemistry, Göttingen, Germany; [2]Institute for Organic and Biomolecular Chemistry, Georg-August-University, Göttingen, Germany

Supplementary Materials and Methods

S1…Synthesis of labeled WALP24

S2…EPR experiments

S3…Molecular modeling of spin labeled WALP24

S4…Orientation selection in WALP24-TOPP experiments and their simulation

Supplementary Figures

Figure S1…Mass spectrometry

Figure S2…CD spectroscopy

Figure S3…Experimental PELDOR/DEER traces in methanol.

Figure S4…Model of WALP24-TOPP for simulation

Figure S5…Orientation selection experiment and simulation series 1

Figure S6…Orientation selection experiment and simulation series 2

Figure S7…Comparison of experiments from series 1 and series 2

Figure S8…PELDOR/DEER experiments on WALP24-MTSSL in DMPC for different peptide:lipid ratios

Figure S9…Echo decay data recorded on WALP24-MTSSL in MeOH and H54-DMPC

Figure S10… Experimental PELDOR/DEER traces recorded in deuterated lipids

Supplementary Materials and Methods

S1 Synthesis of labeled WALP24

Materials and general methods for peptide synthesis

Fmoc-Protected amino acids, coupling reagents and resins were obtained from *GL Biochem* (Shanghai, China), *Iris* (Marktredwitz, Germany), *Acros-Organics* (Geel, Belgium) and *Merck* (Darmstadt, Germany). The amino acid Fmoc-TOPP-OH was synthesized as mentioned in literature (1) The solvents in grades extra dry and puriss. absolute were purchased from *Acros-Organics* (Geel, Belgium), *Sigma Aldrich* (Schnelldorf, Germany) and *Fisher Scientific* (Loughborough, UK). MeOH for HPLC was purchased from commercially available sources and was used as supplied. Water for HPLC was purified using a water purification device from *Millipore* (Bedford, UK). Electrospray ionisation (ESI) mass spectra and high-resolution mass spectra (HR-MS-ESI) were recorded with a *Bruker* maXis spectrometer (Billerica, USA). Microwave assisted SPPS was performed with a *CEM* Discovery microwave instrument (Kamp-Lintfort, Germany). High-performance liquid chromatography was carried out on a *JASCO* instrument (Gross-Umstadt, Germany) using a *Macherey-Nagel* Nucleodur® RP C-18 analytical HPLC column (250 × 4.6 mm, 5 µm). HPLC runs were carried out using a linear gradient of 0.1% aq. TFA (solvent A) and MeOH/0.1% TFA (solvent B) in 30 min with a flow of 1 mL/min.

Loading of the first amino acid. The Fmoc-protected Rink Amide MBHA resin LL/ Fmoc-protected Rink Amide MBHA resin (1.0 eq) was swollen in a BD Discardit II syringe (*Becton Dickinson*, Fraga, Spain) in DMF for 2 h at room temperature and washed with NMP (5×) followed by microwave assisted Fmoc-deprotection with 20% piperidine in DMF (1: 50 °C, 25 W, 30 s; 2: 50 °C, 25 W, 3 min). Between the two deprotection steps the resin was washed with NMP (3×) and afterwards with NMP, DCM, DMF and NMP (10× each). Then a solution of Fmoc-Ala-OH (5.0 eq), 1 hydroxybenzotriazole (HOBt) (5.0 eq) and *N,N'*-diisopropylcarbodiimide (DIC) (5.0 eq) in DMF was added and the coupling was carried out by microwave irradiation (40 °C, 20 W, 10 min). Double coupling was performed. Between the coupling steps the resin was washed with NMP (3×) and after coupling subsequently with NMP, DCM, DMF, MeOH, Et_2O and DCM (5× each) and dried *in vacuo*. The loading density was estimated via UV analysis, followed by capping with 20% Ac_2O in NMP (1.5 mL) at room temperature for 10 min and washing with NMP, DCM, DMF and NMP (10 times each) (2).

General synthesis procedure for the TOPP labeled WALP24. The Fmoc-Ala-preloaded Rink Amide MBHA LL (0.05 µmol, 0.36 mmol/g, 1.00 eq) was swollen in DMF in a 2 mL BD Discardit II syringe for 2 h at room temperature followed by washing with NMP (5×). Each coupling cycle was started by microwave assisted double Fmoc-deprotection by adding 20% piperidine in DMF (1: 50 °C, 25 W, 30 s; 2: 50 °C, 25 W, 3 min) and washing between the deprotection steps with NMP (3×) and afterwards thoroughly with NMP, DCM, DMF and NMP (10 times each). The coupling mixture consisted of the respective amino acid (5.00 eq) in NMP (0.25 mL), a solution of HOBt/*O*-(benzotriazol-1-yl)-*N*,*N*,*N'*,*N'*-tetramethyluronium hexafluorophosphate (HBTU) (5.00 eq/4.90 eq) in DMF (0.5 mL) and a 2 M solution of *N*,*N*-diisopropylethylamin (DIPEA) (10 eq) in NMP (0.25 mL). The mixture was added and the coupling was carried out by microwave irradiation (50 °C, 25 W, 10 min). Double coupling was performed and the resin was washed between the coupling steps with NMP (3×) and afterwards successively with NMP, DCM, DMF and NMP (10 times each). The amino acid Fmoc-TOPP-OH (2.00 eq) was coupled under inert gas in a flask with the coupling reagents 3-(diethoxyphosphoryloxy)-1,2,3-benzotriazin-4(*3H*)-one (DEPBT) (2.00 eq) and $NaHCO_3$ (2.00 eq) in dry THF (1.00 mL) at 0 °C for 4.5 h and room temperature for 30 min. After coupling the resin was washed with NMP, DCM, MeOH, Et_2O and DCM (10 times each). Double coupling and capping with 20% Ac_2O in NMP (1.5 mL) at room temperature for 10 min were performed followed by washing with NMP, DCM, DMF and NMP (10 times each). The following amino acids were coupled using standard conditions as mentioned above. Upon completion of the sequence the resin, was dried *in vacuo*.

General synthesis procedure for the cysteine mutated WALP24. The Fmoc-Ala-preloaded Rink Amide MBHA (1.00 µmol, 0.57 mmol/g, 1.00 eq) was swollen in DMF for 2 h at room temperature. The chain elongation was performed using a microwave assisted automatic peptide synthesizer LibertyTM (*CEM Corporation*, Matthews, NC). Each coupling cycle started with double Fmoc-deprotection with a 20% piperidine solution in NMP (1:50 °C, 25 W, 30 s; 2: 50 °C, 25 W, 3 min) followed by the coupling step. Each amino acid was activated by HOBt/HBTU (5.00 eq/4.90 eq) and DIPEA (10 eq). Double coupling was performed with microwave irradiation (75 °C, 25 W, 5 min). Cysteine was coupled at a lower temperature (50 °C, 25 W, 5 min). After synthesis the resin was dried *in vacuo*.

Cleavage and post-cleavage work up. The peptide cleavage from the resin and simultaneous deprotection of the protecting groups were performed at room temperature for 2 h in a mixture of

- TFA/H_2O/TIS (95/2.5/2.5, *v/v/v*) for the TOPP labeled peptide
- TFA/H_2O/EDT/TIS (94/2.5/2.5/1, *v/v/v/v*) for the cysteine mutated peptide.

After cleavage, the resulting solution was concentrated in a nitrogen stream and the addition of ice-cold Et_2O led to precipitation of the peptide. The resulting suspension was centrifuged at -5 °C followed by decanting the supernatant and washing of the peptide pellet with ice-cold Et_2O (3×). The raw peptide was dried *in vacuo*.

Reoxidation of the TOPP label. The raw peptide (1.00 eq) was dissolved in MeOH (100 µL for 2 mg), $Cu(OAc)_2$ (3.00 eq for each TOPP label) was added and the resulting mixture was stirred 2 h at room temperature followed by purification via HPLC.

Insertion of the MTSSL label. The raw cysteine mutated peptide (1.00 eq) was dissolved in MeOH (100 µL for 2 mg) and MTSSL (3.00 eq for each cysteine) was added. The resulting mixture was shaken over night at room temperature and then purified by HPLC.

TOPP labeled peptide. The following amino acids were used for the peptide synthesis: Fmoc-Trp(Boc)-OH, Fmoc-Lys(Boc)-OH, Fmoc-Ala-OH and Fmoc-Leu-OH. The desired peptide was obtained as a white solid after reoxidation of the TOPP label, purification by HPLC and lyophilisation.
Analytical data: HPLC (analytical, gradient 80 → 100% B in 30 min): t_R = 23.05 min. *m/z* (ESI) = 1023.5 $[M+3H]^{3+}$, 1534.8 $[M+2H]^{2+}$. *m/z* (HR-ESI-MS) = calculated: 1023.5777 $[M+3H]^{3+}$, found: 1023.5781 $[M+3H]^{3+}$ (Fig. S1).

MTSSL labeled peptide. The following amino acids were used for peptide synthesis: Fmoc-Trp(Boc)-OH, Fmoc-Lys(Boc)-OH, Fmoc-Cys(Trt)-OH, Fmoc-Ala-OH and Fmoc-Leu-OH. The desired peptide was obtained after labeling, purification by HPLC and lyophilisation as a white solid.
Analytical data: HPLC (analytical, gradient 80 → 100% B in 30 min): t_R = 24.20 min. *m/z* (ESI) = 753.7 $[M+4H]^{4+}$, 1004.2 $[M+3H]^{3+}$, 1505.8 $[M+2H]^{2+}$. *m/z* (HR-ESI-MS) = calculated: 1004.2170 $[M+3H]^{3+}$, found: 1004.2181 $[M+3H]^{3+}$ (Fig. S1).

CD experiments. The peptide helical structure was investigated by CD spectroscopy in MeOH and in POPC or DMPC lipid vesicles. The measurement in solution was performed with peptide concentrations of 0.03 mg/mL for the TOPP labeled peptide and 0.1 mg/mL for the MTSSL labeled peptide. The experiments in lipids were performed with a peptide/lipid ratio of 1/30 in a 50 mM sodium phosphate buffer (pH 7.5) and peptide concentration of 0.03 mg/mL for the TOPP labeled peptide and 0.05 mg/mL of the MTSSL labeled peptide. For the preparation of the SUV, solutions of the peptide in MeOH and the lipid in $CHCl_3$ were mixed followed by removing of the solvents in a nitrogen stream. Then TFE was added and also removed in a nitrogen stream. The resulting lipid film was dried overnight *in vacuo* at 40 °C. The buffer was added and the film swollen for 30 min at room temperature followed by vortexing the mixture for 1 min at 5 min intervals (3×). To form SUV the mixture was treated with ultrasound sonifier sonoplus HD2076 (*Bandelin*, Berlin, Germany; 30 min, Cycle 4, 60% power). CD spectra are displayed in Fig. S2.

S2 EPR experiments

Determination of labeling efficiency. CW-EPR spectroscopy at X-band frequencies was employed to characterize the label efficiency of WALP24-MTSSL samples. Room temperature experiments were performed in a Bruker Elexsys E500 spectrometer equipped with a Bruker super-high Q resonator ER4122SHQE. Glass capillaries of 1 mm inner diameter (ID) were filled with a sample volume 20 µl. Spin concentrations were calculated by doubly integrating the CW-EPR spectrum and comparing the intensity with a calibration curve recorded with 4-hydroxy-TEMPO at concentrations between 5 and 100 µM as well as with the nominal WALP24-MTSSL concentration determined by UV absorbance of the tryptophans. Labeling efficiencies around 80-90 % were determined for different batches. As the determination of WALP24-TOPP`s concentration is potentially compromised by the interference of TOPP`s absorption spectrum in the WALP24 with the tryptophans, the labeling efficiency can be alternatively estimated from a comparison of the modulation depths of experiments on WALP24-MTSSL and WALP24-TOPP. The software DeerAnalysis offers a tool to calibrate experimental modulation depths to a known labeling efficiency following the relation between the average number of spins per molecule

$\langle n \rangle$ (n = 2 = 100% labeling efficiency), the modulation depth parameter λ (dependence on excitation position, length, and flip angle of the pump pulse) and the total modulation depth Δ: $\Delta = 1 - [\lambda(\langle n \rangle - 1)]$.(3) A comparison of the modulation depths observed in the PELDOR/DEER experiments on WALP24 in MeOH, suggests a labeling efficiency of 65-75 % for WALP24-TOPP. However, we note that this is an estimation as no modulation depth calibration was performed.

labeling efficiency WALP24-MTSSL determination via cw-EPR (experimental modulation depth = 28 %)	labeling efficiency WALP24-TOPP estimation (experimental modulation depth = 16.5 %)
100 % (2 spins)	77.5 % (1.55 spins)
80 % (1.6 spins)	66.5 % (1.33 spins)

Despite the results obtained in the MS spectra, the labeling efficiency of WALP24-TOPP was lower than for WALP24-MTSSL. It seems that the TOPP label is more labile towards reduction, as the spin concentration in the sample was decreasing during the sample preparation procedure.

EPR sample preparation. For experiments in solution, WALP24-TOPP and WALP24-MTSSL were dissolved in MeOH containing 20 % glycerol yielding a spin concentration of 40 µM. EPR samples of WALP24-TOPP and WALP24-MTSSL in deuterated phospholipid multilamellar vesicles (MLV`s) were prepared by mixing stock solutions of the peptide in methanol and the deuterated phospholipid (*Avanti*) in chloroform, yielding a spin concentration of 40 µM (20 µM peptide if labeling efficiency = 100 %) in the final sample volume and a spin:phospholipid molar ratio of 1:3000 (peptide:phospholipid = 1:6000 if labeling efficiency = 100 %). Solvents were removed under nitrogen gas stream followed by 3-4 h vacuum drying. Tris-HCl buffer (20 mM, pH = 7.4) was added to the peptide lipid film and the resulting suspension was mixed on a vortex mixer for 2 min above the lipids transition temperature. Three freeze-thaw cycles were employed to increase the homogeneity of the vesicles. After 30 min of incubation ~ 50 µL were mounted into an EPR tube (3 mm o.d., 2mm i.d) and rapidly frozen in liquid nitrogen. Additionally samples of WALP24-MTSSL in DMPC with different peptide:lipid ratios were prepared (Fig. S8) by mixing stock solutions of the peptide in methanol and the phospholipid (*Avanti*) in dichloromethane, yielding a peptide concentration of 30 µM in the final sample volume and the desired peptide:phospholipid molar ratio. Tris-HCl buffer (20 mM, pH = 7.4) was added to the peptide lipid film and the resulting suspension was mixed on a vortex mixer for 2 min above the lipids transition temperature. After incubation 15 µL were transferred into an EPR tube (2 mm o.d., 1.5 mm i.d) and rapidly frozen in liquid nitrogen. For comparison 15 µL WALP-MTSSL in MeOH containing 20 % glycerol in an EPR tube (2 mm o.d., 1.5 mm i.d) were prepared.

PELDOR/DEER and echo decay experiments. EPR distance measurements were performed using a commercial Bruker ElexSys E580 pulse X/Q-band spectrometer equipped with a pulsed 170 W Q-band TWT-amplifier (Model 187Ka, Applied Systems Engineering Inc.). All PELDOR/DEER experiments were performed using the four-pulse DEER sequence. The experimental temperature of 50 K was achieved with a continuous flow cryostat (CF95550, Oxford Instruments). Experiments on WALP24 in EPR tubes of 3 mm outer diameter (2 mm inner diameter) were performed with the Bruker ER5107QT-II resonator giving a typical π-pulse length of 16-18 ns when frequency was tuned in the dip center of the microwave resonator and 26-28 ns when shifted 90 MHz. The pumping frequency was set in the resonator dip center and at the maximum of the nitroxide`s EPR spectrum, detection frequency was shifted 90 MHz lower. Time delay between the first two pulses in the sequence was set to 400 ns. The dipolar evolution time T (spacing between the first Hahn echo and third detection pulses) was set to 1.9 µs, the last 400 ns of the traces usually contained artifacts and were not considered. Typical acquisition times were on the order 10 to 15 h. Experiments on WALP24-MTSSL in MeOH and DMPC at different peptide:lipid ratios were conducted with smaller sample volumes. For tubes of 1.5 mm outer diameter (1 mm inner diameter) the EN5107D2 Bruker resonator was employed. This resonator delivered a typical π pulse length of 12 ns at the center of the dip and 20-24 ns when the frequency is shifted 90 MHz. The pumping frequency was set in the resonator dip center and at the maximum of the nitroxide's EPR spectrum, while detection frequency shifted 90 MHz lower. Time-delay between the first two pulses in the sequence was set to 300 ns. The dipolar evolution time T (spacing between the first Hahn echo and third detection pulses) was set to 1.4 µs and 0.7 µs for the ratio of 1:250, the last 200 ns of the traces usually contained artifacts and were not considered. Acquisition times were on the order 20 h. For data analysis, dipolar traces were background corrected using either a mono-exponential or a second-order polynomial function. Distance distributions were obtained with the program DeerAnalysis(3), using a fitting procedure based on Tikhonov regularization. The echo decay data on this sample set were recorded with the two-pulse Hahn echo sequence with a systematic increase of the spacing between the two pulses from 300-16636 ns. Detection was set to the maximum of the EPR line.

S3 Molecular modeling of spin labeled WALP24

For the MTSSL containing peptide, an energy minimization of the peptide structure containing the cysteine mutation was performed using PEP-FOLD (5) with subsequent attachment of possible rotamers of MTSSL calculated using MMM (6). For WALP24-TOPP, an optimized WALP24 peptide structure was computed by inserting a tyrosine at the label position to account for possible aromatic π-interaction by the TOPP label. In the geometry optimized structure the tyrosine was subsequently replaced by TOPP, with the TOPP structure as minimized earlier (1).

S4 Orientation selection in WALP24-TOPP experiments and simulation

To investigate the orientation selection expected for the TOPP label a series of experiments and simulations were performed. In order to evaluate the dependence of the dipolar frequency on the experimental set up, experiments were performed with a constant spectral position for pump frequency while varying the frequency separation and therefore only the detection position (series 1 and 2, Fig. S5 and S6). Simulations were conducted using a home written program that takes into account orientation selection in PELDOR experiments (4). Experimental parameters (pulse lengths, frequency separation, EPR detection frequency, nitroxide EPR parameters) were considered in the simulation. Relative orientation of the two TOPP labels was extracted from the minimized WALP24-TOPP structure and the experimental distance. To take into account the rotational freedom of the TOPP label around its two single bonds, Φ_1 and Φ_2 (defined in Fig.1, main text), the simulations were conducted by rotating around the x-axis of the g- and A-tensor which is parallel to the respective single bonds (Fig. S4). A rotation of ± 25° for Φ1 +Φ2 to average orientation selection (as observed in our earlier study(4)) was used to examine the difference in the orientation selectivity as compared to the case ± 0°. Interestingly, there is no distinguishable difference between these two cases (Fig. S5 and S6). The results illustrate that orientation selection is not observed when broadband excitation is applied.

Supplementary Figures

Figure S1: Mass spectra of WALP24.

Up: ESI mass spectrum of the TOPP labeled peptide including the HR-MS-ESI of the $[M+3H]^{3+}$-species as inset. Down: ESI mass spectrum of the MTSSL labeled peptide including the HR-MS-ESI of the $[M+3H]^{3+}$-species as inset.

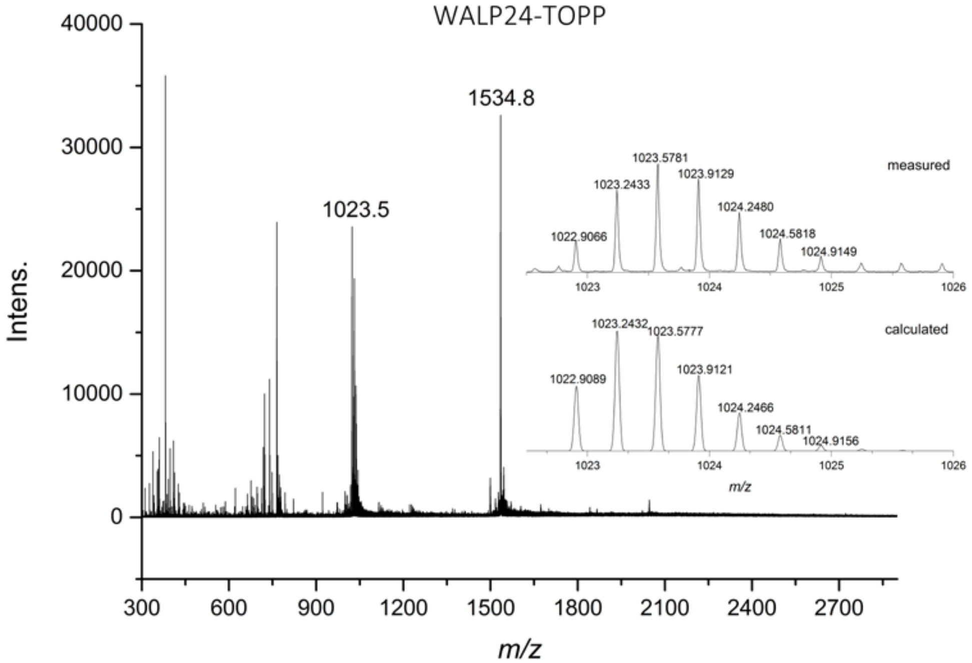

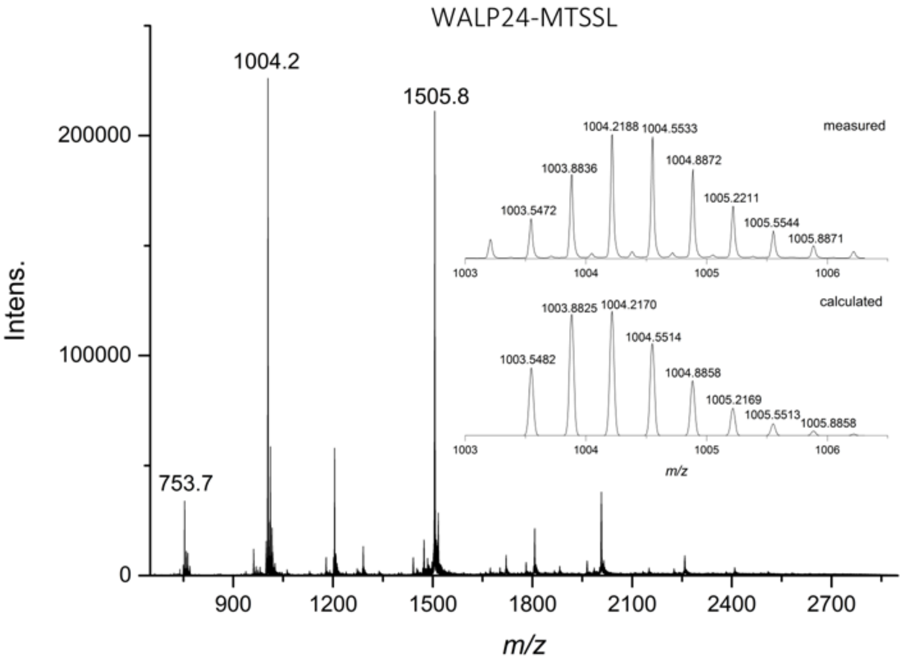

Figure S2: CD spectra of WALP24.

Two characteristic minima at 208 nm and 222 nm indicate α-helical structure. Left: WALP24-TOPP measured in MeOH (black line) and in lipids (colored lines. right; WALP24-MTSSL measured in MeOH (black line) and in lipids (colored lines).

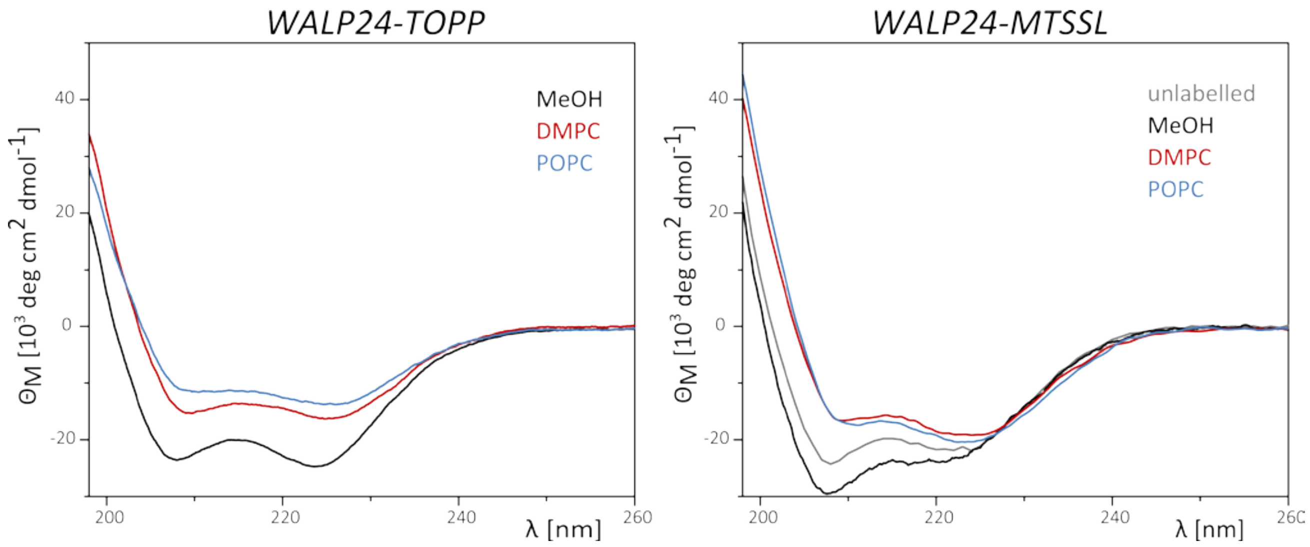

Figure S3: Experimental PELDOR/DEER traces in methanol

A: Experimental time traces of WALP24-TOPP (left) and WALP24-MTSSL (right) recorded in methanol solution.

B: Comparison of distance distribution for WALP24-MTSSL in methanol obtained with Tikhonov regularization using DeerAnalysis (3) (left) with Fourier transformation of the data (right). To test whether the peaks in distance distribution delivered by Tikhonov regularization were real, we have calculated the FT spectrum for each peak and compared it to the FT spectrum of the data. The experimental FT spectrum is well consistent with the sum of at least two distance distributions as given by DeerAnalysis (red and blue curves).

C: Estimation of the uncertainty of the peak distance in the PELDOR traces of WALP24-TOPP in methanol. Illustrated is the expected, simulated (red lines, right), dipolar oscillation as a function of a shift in the peak distance up ± 0.1 nm (left). A Gaussian distance distribution of half width Δ*r* = ± 0.2 nm was considered for the peak distance (red lines, left) with an added noise of the size of the experimental data. It becomes clear that, for the given S/N, a shift of ≥ 0.5 Å in the peak distance is detectable.

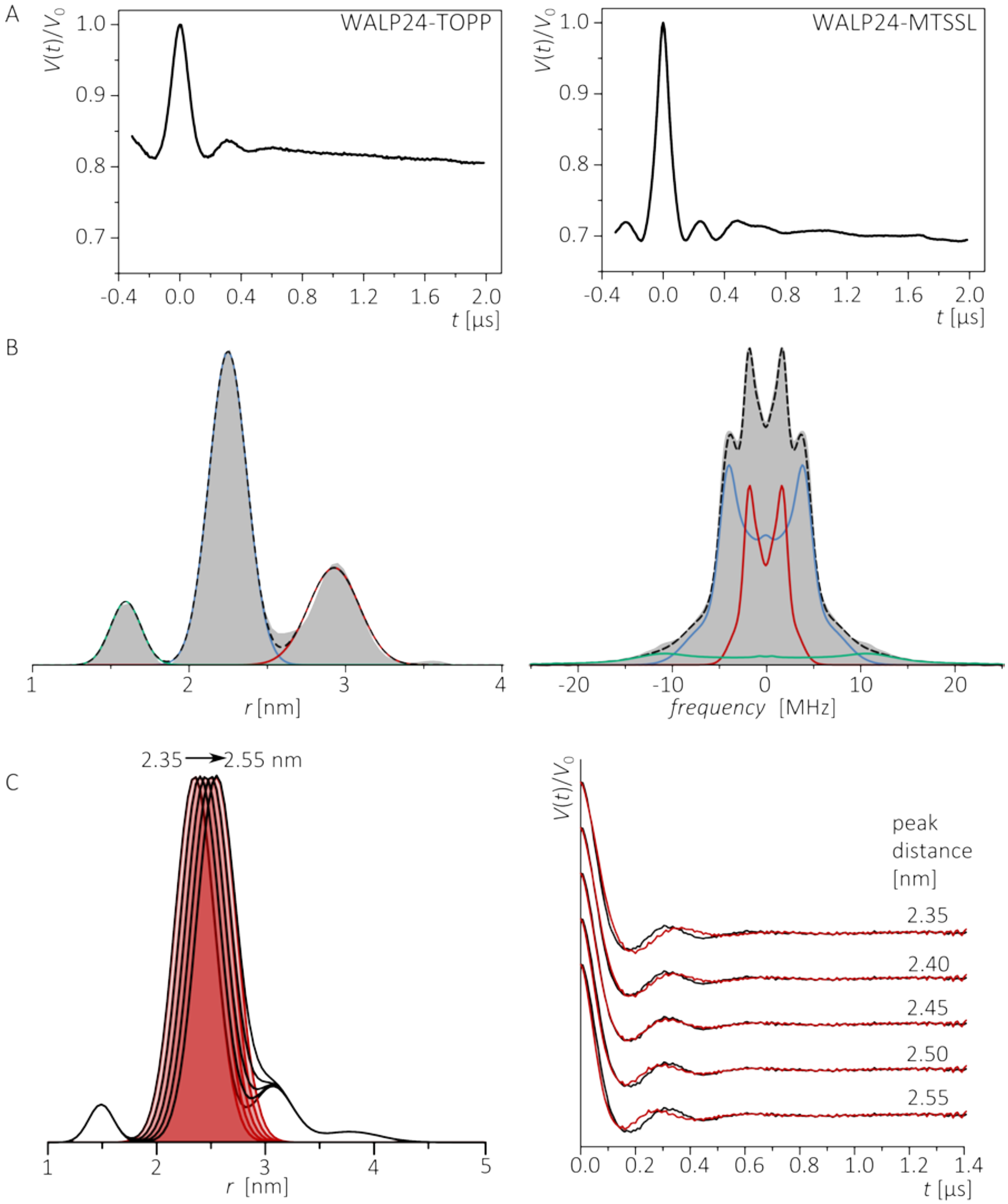

Figure S4: Model of WALP24-TOPP for simulation

Chemical structure of TOPP and orientation of the magnetic g- and A-tensor, which are collinear in the nitroxide radical (left), g- and A-tensor as published in (4). Pymol Model of TOPP attached to minimized WALP24-Y (X = Y. TOPP label at position 5 (label 2) and position 20 (label 1) rotated ± 25° around one of the single bonds illustrating the rotation around the g-tensors x-axis. Methyl-groups and oxygen are hidden for simplicity.

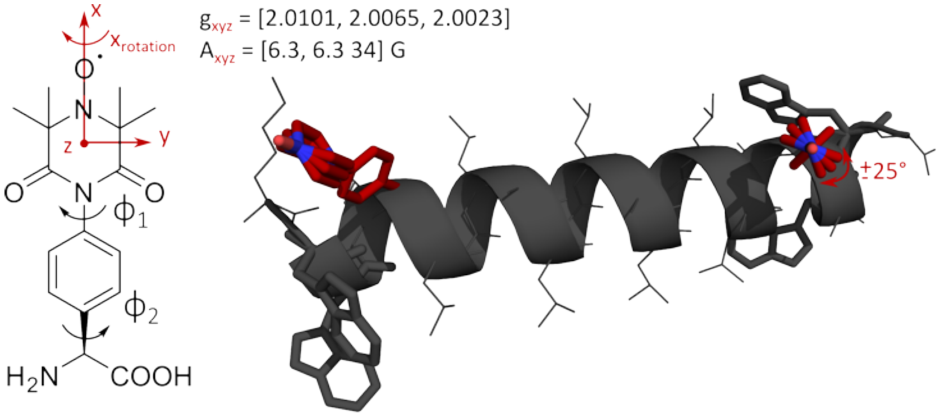

Figure S5: Orientation selection experiment and simulation series 1

A: Experimental set up series 1: experimental ESE (black line), and pulses according to the experimental set up (colored) with the pump pulse applied at the maximum of the EPR spectrum. Frequency separation between pump and detection was varied from 70 MHz to 130 MHz. Pulse lengths were 12 and 18-28 ns for pumping and detection respectively.

B: Fits obtained by Tikhonov regularization performed with DeerAnalysis (left, colored lines) of the experimental time traces (left, black lines), their Fourier transformations (middle) and corresponding distance distributions (right). Experimental time traces and distance distributions show no dependence on the experimental set up.

C: Orientation selective experiments and simulations series 1. Top: experimental (left) and simulated time traces (for rotation around the x-axis of 0° (middle) and ± 25° (right)) for the four set ups of series 1. Bottom: Time traces and corresponding Fourier transformations (inset). The black line, plotted on top of orientation selective simulations (middle, right), displays a simulation with full excitation of the nitroxide`s EPR line for comparison, but no significant differences are observed. Minor differences between the PELDOR/DEER signals simulated with experimental conditions or full excitation of the EPR line are not resolved in the experiment (Fig. S7).

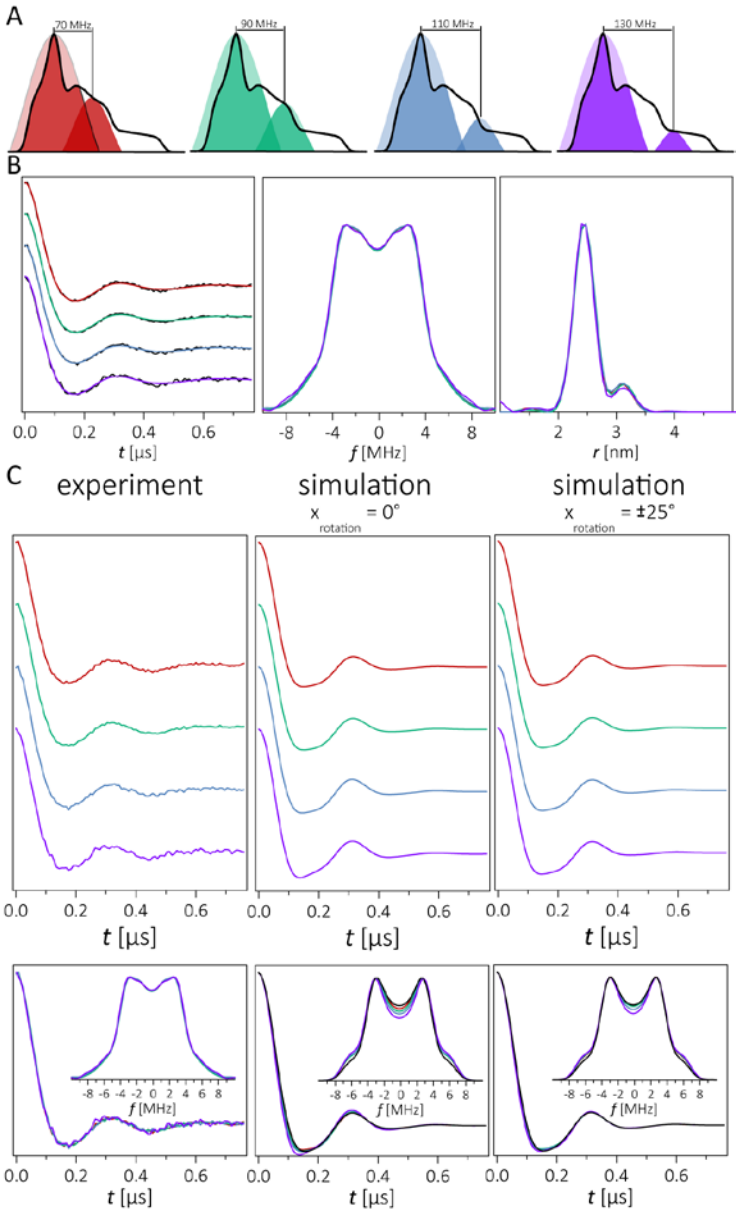

Figure S6: Orientation selection experiment and simulation series 2

A: Experimental set up series 2: experimental ESE (black line), and pulses according to the experimental set up (colored) with the pump pulse applied 10 G below the maximum of the EPR spectrum. Frequency separation between pump and detection was varied from 70 MHz to 130 MHz. Pulse lengths were 12 and 18-28 ns for pumping and detection respectively.

B: Fits obtained by Tikhonov regularization performed with DeerAnalysis (left, colored lines) of the experimental time traces (left, black lines), their Fourier transformations (middle) and corresponding distance distributions (right). Experimental time traces and distance distributions show no dependence on the experimental set up.

C: Orientation selective experiments (left) simulations series 2. Top: experimental (left) and simulated time traces (for rotation around the x-axis of 0° (middle) and ± 25° (right)) for the four set ups of series 2. Bottom: Time traces and corresponding Fourier transformations (inset). The black line, plotted on top of orientation selective simulations (middle, right), displays a simulation with full excitation of the nitroxide`s EPR line for comparison, but no differences are observed. The results best illustrate the absence of orientation selective effects with broadband excitation.

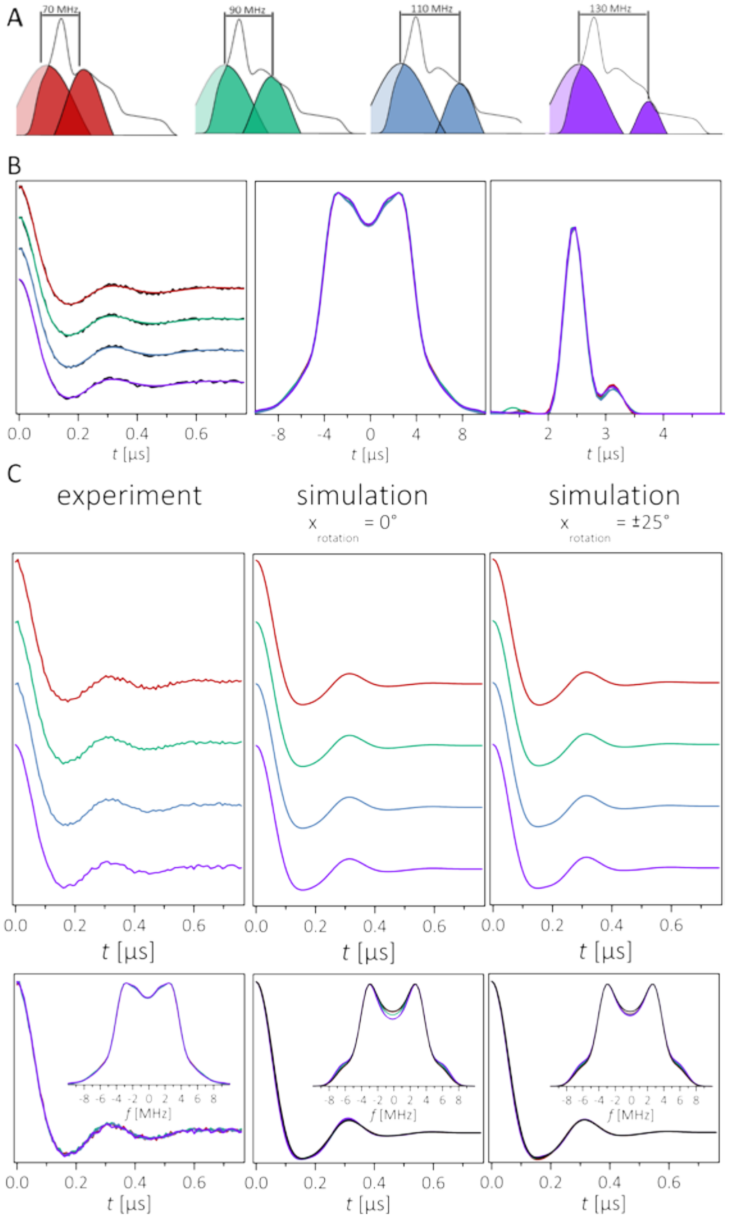

Figure S7: Comparison of experiments from series 1 and series 2

A: Experimental set up: experimental ESE (black line), and pulses according to the experimental set up. Frequency separation between pump and detection was 90 MHz. Pulse lengths were 12 and 18 ns for pumping and detection respectively.

B: Orientation selective simulations series for PELDOR/DEER experiments with 90 MHz frequency separation and pump position either at the maximum of the EPR line (black) or set 10 G lower (gray). Only subtle differences are observed comparing both simulations. A simulation with full excitation of the EPR line is shown in red.

C: Fits of the time traces with Tikhonov regularization (lines, left) of the experimental time traces (dots, left), their Fourier transformations (middle) and corresponding distance distributions (right). Experimental time traces and distance distributions show no dependence on the experimental set up. The subtle differences in simulations for both set ups are not resolved in the experiments. The set up employing the pump pulse at the maximum of the EPR line was chosen for all experiments as it delivers higher modulation depths and higher signal-to-noise ratios.

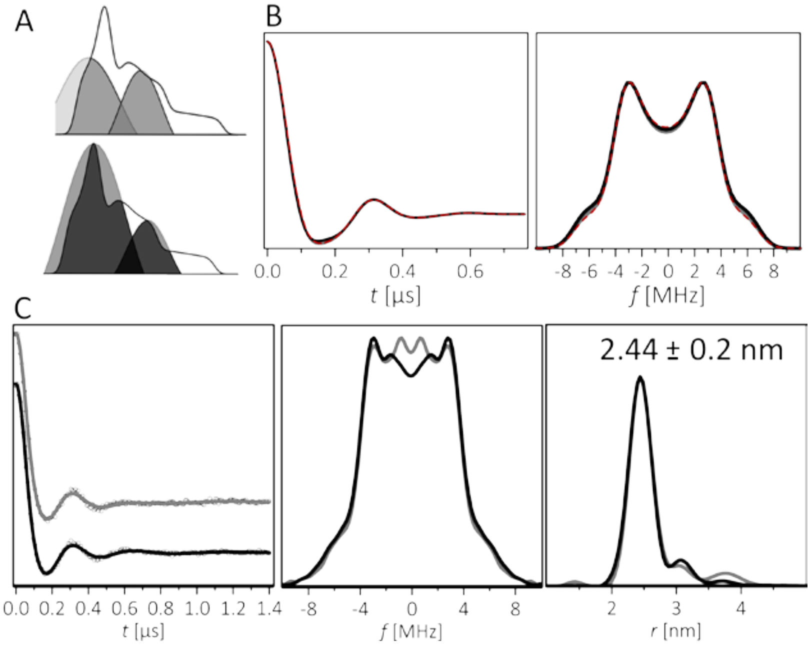

Figure S8: PELDOR/DEER experiments on WALP24-MTSSL in DMPC (protonated) for different peptide-to-lipid ratios.

Experimental PELDOR/DEER traces (left), dipolar traces after background subtraction and their total modulation depth Δ (definition in S2) observed in the experiments (right). Modulation depth for the 1:250 peptide-to-lipid ratio appears much more reduced than in the other samples. However we note that the Δ value in this sample has a larger uncertainty due to the reduced length of the trace (left) and the subsequent difficulty of performing a background subtraction. Nevertheless, a clear trend in increasing echo decay is observed from 1:250 -> 1:3000 peptide-to-lipid ratio, as reported in the main text.

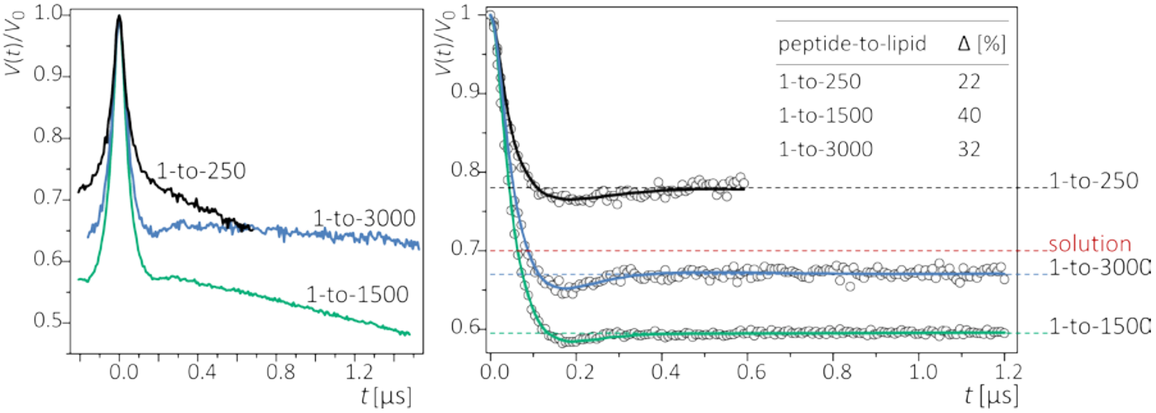

Figure S9: Representative two-pulse echo decay data at Q band recorded on WALP24-MTSSL in MeOH and H54-DMPC.

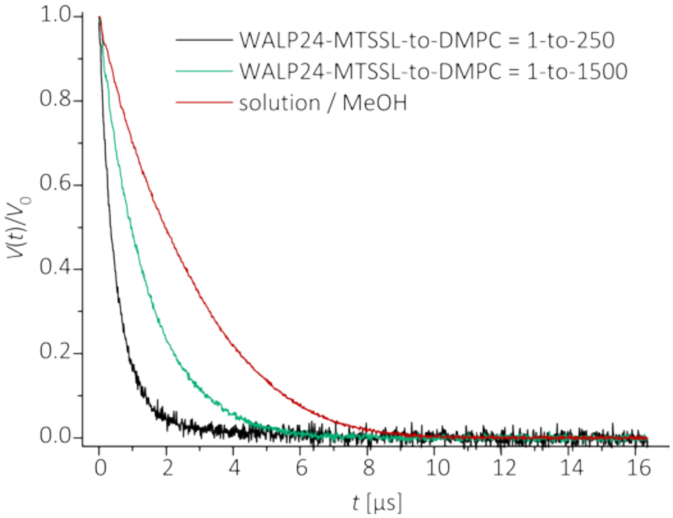

Figure S10: Experimental PELDOR/DEER traces recorded on WALP24-TOPP (left) WALP24-MTSSL (right) in deuterated lipids D54-DMPC (red) and D31-POPC (blue) prior background subtraction.

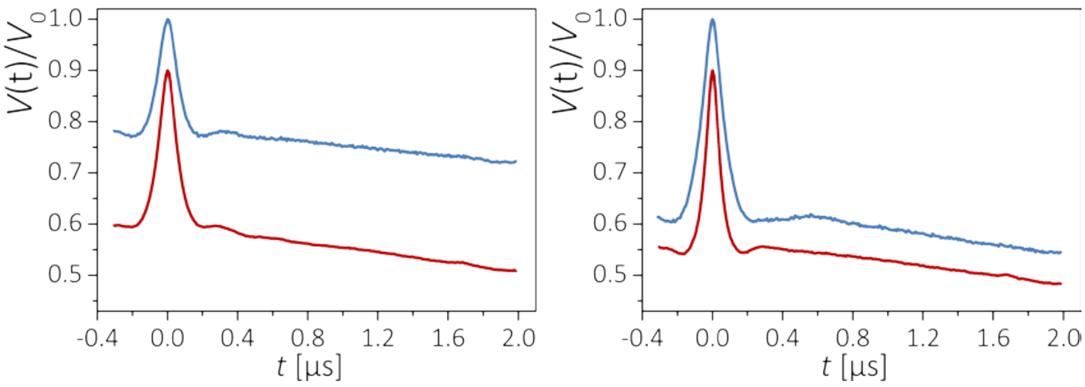

REFERENCES

1. Stoller, S., G. Sicoli, T. Y. Baranova, M. Bennati, and U. Diederichsen. 2011. TOPP: a novel nitroxide-labeled amino acid for EPR distance measurements. Angewandte Chemie 50:9743-9746.
2. Gude, M., J. Ryf, and P. D. White. 2002. An accurate method for the quantitation of Fmoc-derivatized solid phase supports. Letters in Peptide Science 9:203-206.
3. Jeschke, G., V. Chechik, P. Ionita, A. Godt, H. Zimmermann, J. Banham, C. R. Timmel, D. Hilger, and H. Jung. 2006. DeerAnalysis2006—a comprehensive software package for analyzing pulsed ELDOR data. Applied Magnetic Resonance 30:473-498.
4. Tkach, I., S. Pornsuwan, C. Hobartner, F. Wachowius, S. T. Sigurdsson, T. Y. Baranova, U. Diederichsen, G. Sicoli, and M. Bennati. 2013. Orientation selection in distance measurements between nitroxide spin labels at 94 GHz EPR with variable dual frequency irradiation. Physical chemistry chemical physics : PCCP 15:3433-3437.
5. Shen, Y., J. Maupetit, P. Derreumaux, and P. Tuffery. 2014. Improved PEP-FOLD Approach for Peptide and Miniprotein Structure Prediction. Journal of chemical theory and computation 10:4745-4758.
6. Polyhach, Y., E. Bordignon, and G. Jeschke. 2011. Rotamer libraries of spin labelled cysteines for protein studies. Physical chemistry chemical physics : PCCP 13:2356-2366.
7. Bode, B. E., D. Margraf, J. Plackmeyer, G. Dürner, T. F. Prisner, and O. Schiemann. 2007. Counting the Monomers in Nanometer-Sized Oligomers by Pulsed Electron−Electron Double Resonance. Journal of the American Chemical Society 129:6736-6745.
8. Dastvan, R., B. E. Bode, M. P. R. Karuppiah, A. Marko, S. Lyubenova, H. Schwalbe, and T. F. Prisner. 2010. Optimization of Transversal Relaxation of Nitroxides for Pulsed Electron−Electron Double Resonance Spectroscopy in Phospholipid Membranes. The Journal of Physical Chemistry B 114:13507-13516.
